# The outbreaks of nontarget mirid bugs promote arthropod pest suppression in Bt cotton agroecosystems

**DOI:** 10.1111/pbi.13233

**Published:** 2019-08-27

**Authors:** Wenjing Li, Lili Wang, Coline C. Jaworski, Fan Yang, Bing Liu, Yuying Jiang, Yanhui Lu, Kongming Wu, Nicolas Desneux

**Affiliations:** ^1^ State Key Laboratory for Biology of Plant Diseases and Insect Pests Institute of Plant Protection Chinese Academy of Agricultural Sciences Beijing China; ^2^ Department of Zoology University of Oxford Oxford UK; ^3^ Aix‐Marseille University CNRS IRD Univ. Avignon Marseille France; ^4^ National Agro‐Technical Extension and Service Center Beijing China; ^5^ INRA CNRS UMR‐ISA Université Côte d'Azur Nice France

**Keywords:** Bt crop, nontarget pest, ecological effect, pest status evolution, pest management, mirid bug, aphid

The adoption of Bt (*Bacillus thuringiensis*) crops has improved crop yield, reduced chemical insecticide use and induced an increase in farmer profits; however, some concerns persist about their potential environmental risks, including the impact on nontarget arthropods (Romeis *et al*., 2008). In China, Bt cotton was first grown commercially in 1997. As the levels of cultivated Bt cotton increased, populations of the target pest *Helicoverpa armigera* were found to have substantially declined (Wu *et al*., 2008). In addition, reduced insecticide use in Bt cotton has shown positive side effects, such as increased pest biocontrol services provided by natural enemies (Lu *et al*., 2012), but also negative side effects, such as mirid bug outbreaks (Lu *et al*., 2010). How this shift in pest status may impact interspecific arthropod interactions in Bt cotton needs to be further investigated (Hagenbucher *et al*., 2013; Zeilinger *et al*., 2011; Zhang *et al*., 2018). Mirid bug feeding often causes tattered leaves on host plants, suggesting that interspecific competition with other leaf‐feeding insects may occur. Furthermore, mirid bugs may also prey on the cotton aphid *Aphis gossypii* and other arthropod pests (Jiang *et al*., 2015). Hence, mirid bugs have the potential to act as arthropod biocontrol agents during their outbreaks.

In northern China, *Apolygus lucorum* is the most common mirid bug species in cotton fields. We assessed predation by *A. lucorum* on cotton aphids in Petri dishes and found that predation was high and generally increased with increasing prey density (Figure 1a). In a greenhouse experiment, we further assessed interspecific competition by piercing cotton leaves with an insect needle dipped in the salivary extract of *A. lucorum* to simulate feeding by this species. Cotton plants with simulated plant feeding harboured significantly fewer aphids than control plants, which indicated the presence of evident interspecific competition mediated by plant feeding (Figure 1b). We evaluated the effect of the presence of *A. lucorum* (including predation and interspecific competition; Figure 1c) on cotton aphid dynamics in the greenhouse and field cages. The aphid abundance was significantly reduced in the presence of mirid bugs in both trials (Figure 1d, e). These results provide new evidence that aphid populations are efficiently reduced by *A. lucorum*.

**Figure 1:**
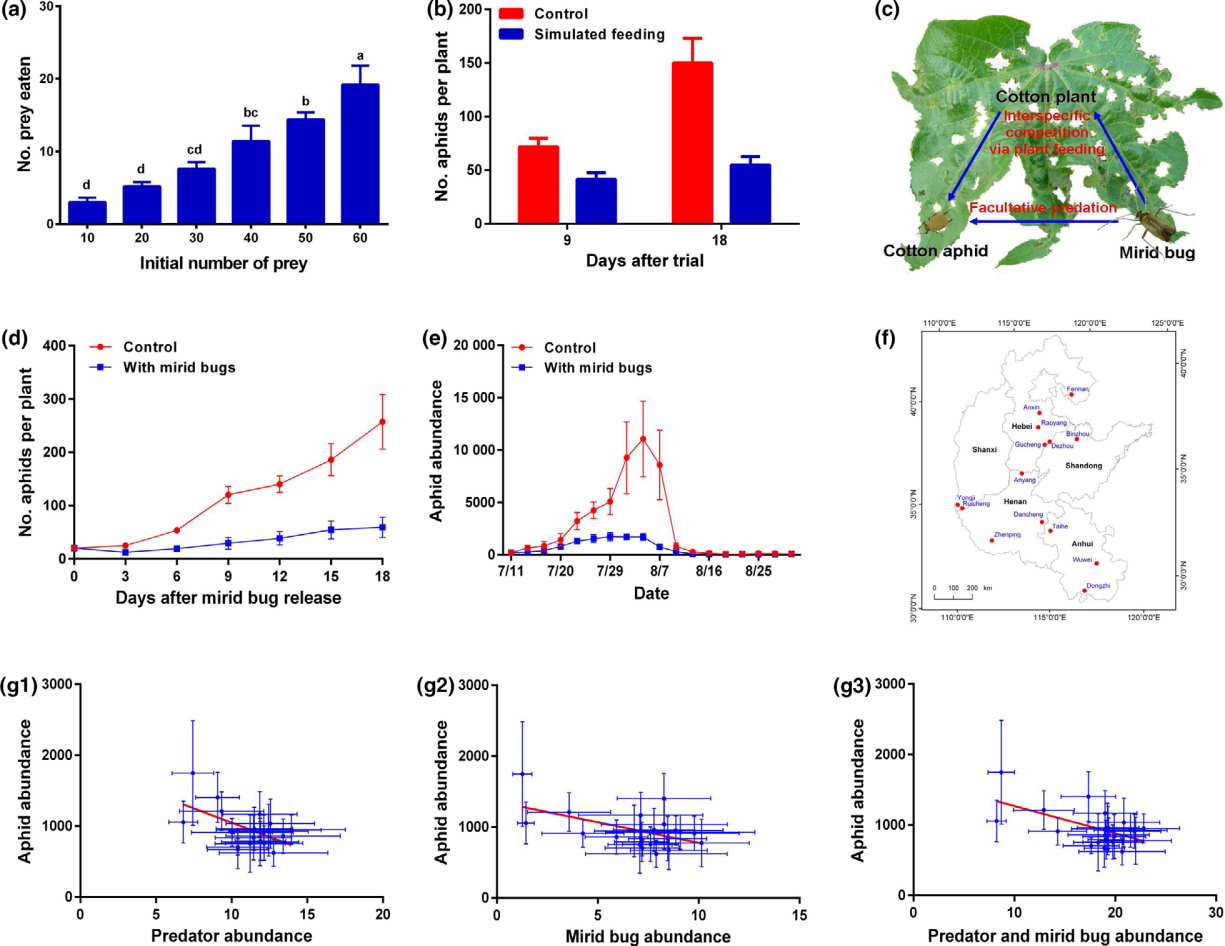
(a) Prey consumption rates of *Apolygus lucorum* on cotton aphids in a Petri dish over 24 h (*F*_5,24_ = 15.79, *P *<* *0.0001). (b) Effects of simulated plant feeding by *A. lucorum* on cotton aphid abundance in the greenhouse (9 days after trial: *t* = 3.00, df = 28, *P *=* *0.0057; 18 days: *t* = 3.90, df = 28, *P *=* *0.0005). In each cage, there was one cotton plant with an initial population of 20 cotton aphids. (c) The interactive effect of mirid bugs and cotton aphids on cotton plants. The cotton leaf in the figure was greatly damaged by mirid bugs. (d) Effects of the presence of *A. lucorum* on cotton aphid population growth in greenhouse cages (*F*_1,9_ = 32.87, *P *=* *0.0003). Before the trial, there was one cotton plant with 20 aphids per cage. (e) Effects of the presence of *A. lucorum* on aphid population increase on cotton plants within field cages (*F*_1,16_ = 8.19, *P *=* *0.0211). There were 10 mirid bugs and 200 cotton aphids on 10 cotton plants per cage, and the control contained 200 aphids without mirid bugs. (f) Locations for long‐term monitoring during 1997–2017 in northern China. (g) Linear relationships between the abundances of generalist predators, mirid bugs and cotton aphids in cotton fields in northern China (1997–2017). (g‐1) Relationship between predator abundance (*x*) and aphid abundance [*y*, log_10_(*n*) transformed]: *y* = −0.07*x* + 7.61, *F*_1,19_ = 6.97, *R* ^2^ = 0.27, *AIC *= −0.65, *P *=* *0.0162. (g‐2) Relationship between mirid bug abundance (*x*) and aphid abundance [*y*, log_10_(*n*) transformed]: *y* = −0.05*x* + 7.19, *F*_1,19_ = 6.81, *R* ^2^ = 0.26, *AIC* = −0.52, *P *=* *0.0173. (g‐3) Relationship between predator and mirid bug abundance (*x*) and aphid abundance [*y*, log_10_(*n*) transformed]: *y* = −0.03*x* + 7.45, *F*_1,19_ = 8.34, *R* ^2^ = 0.31, *AIC* = −1.74, *P *=* *0.0094.

We analysed the relationships of the abundance of generalist predators (ladybeetles, lacewings and spiders), mirid bugs and cotton aphids in cotton fields in northern China from 1997 to 2017 (Figure 1f). Linear regression analyses showed that increasing abundances of mirid bugs and generalist predators were significantly correlated with decreasing aphid abundance, in agreement with the potential suppression of cotton aphids by mirid bugs highlighted above (Figure 1g‐1, g‐2). Moreover, the effect of ‘Predators + Mirid bugs’ explained more variation in the model than the individual effects of these variables based on the *AIC* (smaller is better) and *R* ^2^ values, which suggests that stronger suppression of cotton aphids occurred when mirid bugs served as the biocontrol agents of this pest (Figure 1g‐3). This result indicates that mirid bug outbreaks promote the biocontrol service of aphid suppression provided by generalist predators.

Many species of mirid bugs are omnivorous (Wheeler, 2001). The current study highlights the biocontrol potential of *A. lucorum* against cotton aphids via either predation or plant‐mediated competition. Interspecific competition may occur in these species mainly because mirid bug feeding increases the secondary insect‐resistant compound (e.g. condensed tannin) content in cotton plants and decreases the availability of plant food for the aphids (because the leaves are damaged; Jiang *et al*., 2015). Through the combination of facultative predation and interspecific competition, *A. lucorum* showed the potential to suppress the aphid population in greenhouse and field‐cage trials. Additionally, we showed that the population sizes of mirid bugs were negatively correlated with aphid population sizes in cotton fields over a long period of time and at a regional scale. With the wide‐scale adoption of Bt cotton in China, chemical insecticide use has been greatly reduced (Zhang *et al*., 2018), which further indicates the importance of biocontrol agents (including natural enemies and mirid bugs) in suppressing the aphid populations in Bt cotton fields. In preliminary experiments, we found that *A. lucoru*m and another mirid bug (*Adelphocoris suturalis*) attack a variety of pest species, such as *H. armigera*,* Pectinophora gossypiella*,* Myzus persicae*,* Tetranychus urticae* and *Bemisia tabaci*. Hence, mirid bug outbreaks in multiple crops likely affect the populations of different arthropod pests in Bt cotton agroecosystems. Mirid bugs could be both phytophagous and zoophytophagous, acting as pests or as natural enemies (of other pests), respectively. Therefore in the context of IPM practices, they may be considered as biocontrol agents when their density is below the economic threshold and considered as crop pests when they exceed this threshold.

In combination with those from previous studies, our results provide information regarding a complex case of population status change in several arthropods after Bt cotton adoption in China. Hence, we highlight the critical need for long‐term and landscape‐level risk assessments of transgenic crop use.

## Author contributions

Y.L. and K.W. designed research. W.L., L.W., F.Y., B.L. and Y.J. performed research. W.L., Y.L. and B.L. analysed data. Y.L., W.L., C.J. and N.D. wrote the paper.
